# Biting time of day in malaria mosquitoes is modulated by nutritional status

**DOI:** 10.1101/2025.04.28.650966

**Published:** 2025-05-01

**Authors:** Catherine E. Oke, Samuel S. C. Rund, Maxwell G. Machani, Abdul Rahim Mohammed Sabtiu, Yaw Akuamoah-Boateng, Yaw A. Afrane, Sarah E. Reece

**Affiliations:** 1 Institute of Ecology and Evolution, School of Biological Sciences, University of Edinburgh, Charlotte Auerbach Road, Edinburgh, UK;; 2 Institute of Immunology and Infection Research, School of Biological Sciences, University of Edinburgh, Charlotte Auerbach Road, Edinburgh, UK;; 3 Center for Research Computing, University of Notre Dame, Notre Dame, IN, 46556, USA;; 4 Department of Biological Sciences, University of Notre Dame, Notre Dame, IN, 46556, USA;; 5 Entomology Department, Centre for Global Health Research, Kenya Medical Research Institute, Kisumu, Kenya;; 6 Department of Medical Microbiology, University of Ghana Medical School, College of Health Sciences, University of Ghana, Accra, Ghana.

## Abstract

**Background and objectives:**

Vector-borne disease transmission follows daily rhythms because the transmission of pathogens occur at the time of day vectors forage for blood. Insecticide-treated bed nets significantly reduce malaria transmission by interrupting the host seeking behaviour of *Anopheles* spp. mosquitoes, yet residual transmission is an increasing problem. Biting when humans are unprotected by bed nets is thought to be a driver of residual transmission, but why mosquitoes are shifting their biting rhythms is poorly understood. We test whether food availability, which mediates activity and foraging rhythms across diverse animal taxa, influences the time of day that mosquitoes bite.

**Methodology:**

We varied the amount of blood and sucrose that female *Anopheles gambiae s.l*. received, and used human-mimic traps in a semi-field system to test the hypothesis that low resources cause host seeking to occur at earlier and later times of day.

**Results:**

Nutritional resources determine both the likelihood and time of day that host seeking occurs. Specifically, low-resourced mosquitoes were 2–3 fold more likely to host seek overall, and 5–10 fold more likely to host seek at an earlier time of day than well-resourced mosquitoes, which predominantly sought a host in the second half of the night time.

**Conclusions and Implications:**

By driving plasticity in biting time of day, mosquito nutritional condition is an underappreciated contributor to residual malaria transmission. Understanding the drivers of biting time of day variation, and their impacts on parasite development, is crucial for the future success of vector control tools and controlling malaria transmission.

## Lay summary

We reveal that the nutritional status of mosquitoes influences the time of day that they seek a human to bite. Specifically, poorly-fed mosquitoes attempt to bite humans in the early evening when they are unlikely to be protected by bed nets, which could lead to increased malaria transmission.

## Background and Objectives

The transmission of vector-borne diseases (VBDs), such as malaria and dengue, relies on the foraging rhythms of insect vectors, which take up pathogens from an infected vertebrate host during a blood meal and transmit them to a new host during a subsequent blood-feeding event [1,2]. For example, *Plasmodium* parasites, the causative agent of malaria, are vectored between humans by members of the *Anopheles* genus of mosquitoes, which are generally nocturnally active and preferentially bite between the hours of 11pm and 4am (*i.e*. the ‘classical’ time window) [3–5]. Control strategies, such as insecticide-treated bed nets (ITNs) and indoor residual spraying (IRS), exploit rhythmicity in foraging and resting behaviours, respectably, to bring mosquitoes into contact with a lethal insecticide. Bed nets are one of the most effective methods to curb the spread of malaria [6,7], averting 68% of deaths since 2000 [8]. Despite this success, residual transmission occurs, in part due to the evolution of physiological insecticide resistance (e.g. biochemical and morphological modifications) and behavioural resistance in vector populations [9]. In particular, millions of clinical malaria cases are predicted to be occurring annually due mosquitoes altering their biting time of day [10].

There are increasing reports suggesting that *Anopheles* spp. are shifting their biting time of day to earlier in the evening (‘early’ biting) or later in the morning (‘late’ biting) when humans are unprotected by bed nets [4,5,11–15]. While shifts in biting time of day may promote transmission simply due to increased access to hosts, vector foraging at non-classical times of day affects multiple parameters of parasite fitness and transmission (often expressed as the basic reproductive number, R_0_ [16]) in complex and potentially opposing ways [1]. For example, studies with animal models of malaria reveal that mosquitoes are less susceptible to *Plasmodium* infection at night [17], likely due to rhythmicity in mosquitos’ immune defences [18], but parasites are intrinsically more infective to mosquitoes at night, and both mosquito and parasite rhythms interact to influence parasite productivity within mosquitoes and onwards transmission potential [17,19]. There are also increased risks of desiccation and predation to mosquitoes foraging at suboptimal times of day. Further, energetic costs could result from rhythmic metabolic processes being misaligned with food intake [1]. Therefore, shifts in biting time of day are likely to impact on vectorial capacity and the success of vector control tools in hard to intuit, but important, manners [6].

While *Anopheles* spp. biting rhythms are weakly heritable, variation in biting time of day is predominantly driven by non-genetic factors [20]. This suggests that mosquitoes use phenotypic plasticity to adjust their foraging behaviour in response to environmental conditions [21], such as limited access to blood meals. However, the extent to which mosquitos can shift biting time of day and the range of potential environmental drivers are poorly understood. Variation in physiological condition and resource availability can cause animals to alter their foraging rhythms [22] in adaptive (i.e. fitness enhancing) ways, especially in resource-limiting conditions [23]. For example, low food availability promotes a shift to diurnal foraging patterns in small nocturnal rodents, which minimises energy loss by remaining in burrows during particularly cold nights [24,25]. Similarly, mosquitoes may garner greater benefits from shifting biting time of day when they are in poor nutritional condition. Such context-dependent variation in the costs and benefits of shifts in biting time may explain why biting times have seemingly not significantly changed in some *Anopheles* spp. populations despite high ITN use [26,27], and why the evolution of behavioural resistance varies across populations in manners that correlate with the abundance of sources of nutrition for mosquitoes [28].

The rhythmic feeding ecology of anopheline mosquitoes [13,29,30] is under circadian clock control [31] and revolves around the need for blood and sugar meals. Female mosquitoes utilise proteins and lipids in blood for egg production, and sugar sources power flight and the accumulation of energy reserves [32]. Females take a blood meal after mating, beginning their first gonotrophic cycle of egg development and oviposition, seeking more blood approximately every 3 days to fuel subsequent gonotrophic cycles [33]. The type and abundance of nutritional resources available to mosquitoes varies across habitats and seasons [32,34], and vector control tools such as ITNs can limit blood meal availability [35]. In response, mosquitoes exhibit adaptive plasticity in multiple feeding behaviours; for example, failure to acquire a blood meal leads to increased sugar feeding [30] to promote survival until future biting opportunities. Malaria parasites rely on mosquito host seeking for transmission [36]; they are taken up during a blood meal and become ready to transmit to a new host after approximately 10–14 days, during a subsequent blood meal. However, despite the importance of mosquito foraging rhythms for malaria transmission and evidence that physiological condition can alter foraging rhythms in other taxa, the effect of resource availability on mosquito biting time of day is unknown.

Here, we perturb the nutritional resources provided to adult mosquitoes to test whether they respond by plastically altering the time of day that host seeking occurs. We simulated scenarios in which mosquitoes have taken an initial blood meal (simulating the point at which malaria parasites would be acquired), and then: (i) were unsuccessful at acquiring a subsequent blood meal and only have access to a sugar-poor environment, (ii) were unsuccessful at acquiring a subsequent blood meal but have access to a sugar-rich environment, and (iii) successfully acquired a subsequent blood meal as well as having access to a sugar-rich environment. We reveal that mosquitoes with access to additional blood meals and/or high levels of sugar host seek during the classical night-time biting window and into the early morning (i.e. late biting), but when sugar and blood are limited, mosquitoes are more likely to host seek in the evening (i.e. early biting) when humans are unlikely to be protected by ITNs. We also supplement our data with preliminary field collections that suggest biting time of day of wild-caught adult mosquitoes may correlate with some aspects of nutritional condition. We discuss the potential outcomes of shifts in biting time of day in response to resource availability for understanding residual transmission patterns, and for parasite fitness.

## Methodology

We performed a semi-field experiment to assess if mosquito nutritional status impacts host seeking and biting time of day. We reared wild-caught larvae, and maintained blood fed female *Anopheles gambiae s.l*. mosquitoes from the F_2_ generation on diets that varied in nutritional resources, before releasing them into an enclosed semi-field system with traps mimicking human odour. The traps were programmed to separately capture mosquitoes biting at early (evening), classical (during the night) and late (morning) times of day.

### Mosquitoes

Experimental mosquitoes were F_2_ progeny of wild-caught *Anopheles gambiae s.l*. larvae, collected from three sites across the Greater Accra region of Ghana (Tuba, 5° 30’ 47” N 0° 23’ 16” W; Teshie, 5° 35′ 0″ N, 0° 6′ 0″ W; East Legon, 5° 38’ 16.39” N, 0° 9’ 40.33” W) in Dec 2023-Jan 2024 (Supplementary Fig. S1). We maintained all generations of infection-free mosquitoes under standard rearing conditions (26±2°C, 80% relative humidity with a 12L:12D cycle) with blood provided via direct-feeding [37]. We fed both F_0_ and F_1_ generations *ad libitum* with 10% sucrose solution in water, and provided them with soaked cotton wool in petri dishes for egg laying. On day 1 post-emergence of the F_2_ generation, we transferred female mosquitoes to three experimental cages (n=100–150 per cage) and allocated each cage to one of three feeding treatments: (i) 0.5% sucrose in water (0.5% suc), (ii) 10% sucrose in water (10% suc) or (iii) 10% sucrose in water plus an additional blood meal on day 6 post-emergence (10% suc + bm). We maintained all treatment groups on their feeding treatments from day 1 post-emergence until the host seeking assay. In addition, to mimic mosquito life history in nature, we provided all groups with an initial blood meal on day 3 post-emergence and the opportunity to lay eggs. On day 9–10 post-emergence, we transferred mosquitoes from each cage to paper cups (n=70–100 per cup) and provided them with water only for six hours prior to assaying biting time of day. We reared six batches of mosquitoes to conduct a total of six semi-field system releases.

### Semi-field host seeking experiment

**Figure 1.**
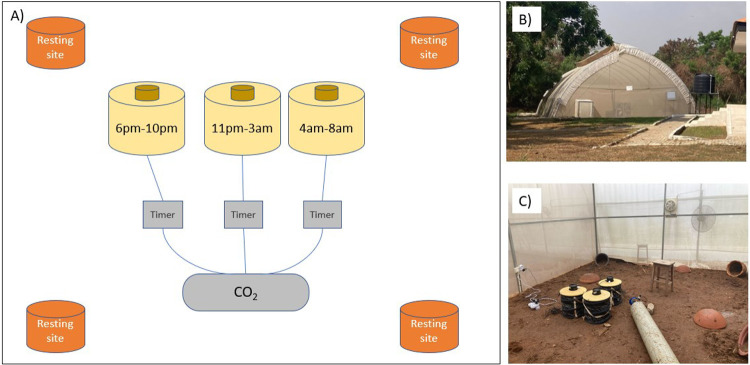
The semi-field behavioural assay set-up. Schematic diagram of the set-up (A), featuring four resting sites for mosquitoes, and traps baited with human odour which were programmed to turn on at 6pm-10pm, 11pm-3am and 4am-8pm respectively. These windows correspond to approx. ZT12-ZT16, ZT17–21, and ZT22-ZT2 respectively, where Zeitgeber time (ZT) defines the hours since lights on and ZT0/24 is dawn. All traps were linked to a CO_2_ source, which was split to three timers ensuring the CO_2_ was only flowing when the trap that was switched on. Photographs show the semi-field facility (B) and the behavioural assay set-up (C).

We conducted mosquito behavioural experiments in January-February 2024 at the University of Ghana in an enclosed semi-field system (the “MalariaSphere”, [38,39]). The average (± SEM) minimum and maximum daily temperatures during the experimental releases were 25.8±0.6°C and 34.5±0.4°C respectively [40]. The enclosure of 5.8 × 4.2 × 2.8m was covered in an insect-proof screen to prevent mosquito escape and/or entry from the external environment. We placed three BG-Sentinel traps (Biogents, Germany) in the enclosure, with an MB5 human-mimic lure developed to attract anopheline mosquitoes (Biogents, Germany) and a CO_2_ flow of approximately 21g/hour, to mimic human scent and breath. We programmed the traps to turn on at 6pm-10pm to capture ‘early’ biters, 11pm-3am to capture ‘classical’ biters and 4am-8am to capture ‘late’ biters (Fig. 1). CO_2_ flow was controlled with BG-CO_2_ timers (Biogents, Germany), ensuring CO_2_ was only emitted when the respective trap was switched on.

Within the MalariaSphere, released mosquitoes could access resting sites (four clay pots around the enclosure). We did not provide water or food sources because this would have confounded the experimental treatment groups. We colour-marked mosquitoes from each feeding treatment using aerosolised fluorescent powder (FTX series, Swada London), applied following Machani et al [38]. We rotated the colour of each treatment group across sequential releases to ensure any differential impacts of colour on mosquito behaviour did not confound treatment groups. We carried out six releases over a period of three weeks, with mosquitoes being released into the enclosure between 5pm-5:30pm, just prior to dusk at 6pm (n=235–300 mosquitoes per release). A period of at least 48 hours between each release and checking all resting sites with a Prokopack aspirator ensured that any remaining untrapped mosquitoes had been removed or died before the next release. We collected mosquitoes from traps at 8am the day after the release, then cold-anaesthetised and counted them.

### Nutrition assays

Immediately prior to each release, we collected a subset of mosquitoes from each feeding treatment group (n=14–19 per group, total n=48) to confirm our resource perturbations impacted nutritional status. These mosquitoes were frozen at −20°C for lipid, glycogen and total sugar analyses using modified Van Handel protocols [41–43]. Firstly, we removed a wing from each mosquito to quantify body size. Wing length is a commonly used proxy for adult body size because dry weight and wing length are highly correlated [44]. We then lysed individual mosquitoes in 100μl 2% sodium sulphate and added 750μl of 1:2 chloroform:methanol. We centrifuged samples at 12000 rpm for 3 minutes; the supernatant was used for lipid and total sugars analysis, and the precipitate was used for glycogen analysis. We conducted lipid, glycogen and total sugars assays following Oke et al [45].

### Statistical analysis

We used R v. 4.1.3 to perform data analysis. We confirmed that adult body size did not differ across the treatments using a linear model with wing length, mosquito release batch and their interaction as main effects (Supplementary Fig. S2). We then analysed nutritional content (lipid, glycogen, total sugars) of individual mosquitoes using linear mixed models with feeding treatment as a main effect and mosquito release batch as a random effect, accounting for any within treatment variation in body size. To investigate how feeding treatments impacted the overall proportion of mosquitoes caught across each release session, we used binomial generalised linear mixed models (glmm) with mosquito release batch as a random effect and feeding treatment, trap time and their interaction as main effects. We estimated proportion of caught mosquitoes and relative odds ratios (OR) ± SE from models using the *emmeans* [46] package. We square root transformed nutrition data to meet assumptions of normality and homogeneity of variance. We minimised all models using likelihood ratio tests and AICc for non-nested models, and confirmed model assumptions using the *easystats* [47] package. To account for differences between mosquito release batches, we present estimated marginal means ± SEM (*emmeans* package), predicted from models. Whenever the minimised model contained a significant effect of feeding treatment, we conducted post hoc pairwise comparisons using the Tukey method with the *emmeans* package.

### Ethics statement

The study protocols for mosquito colony blood feeds and human landing catches (HLCs) (see Discussion and Supplementary Methods) were ethically reviewed and approved by the Ghana Health Service Ethics Review Committee (GHS-ERC: 021/07/23). Volunteers involved in colony blood feeds were tested for malaria prior to direct-feeding, and the same person fed the mosquitoes throughout the experiment. Permission to conduct HLCs in study sites was obtained from the District Assembly Representative, and written consent was obtained from all collectors. Collectors had access to malaria prophylaxis and did not report any adverse events.

## Results

### Resource availability perturbs mosquito nutritional status

**Figure 2.**
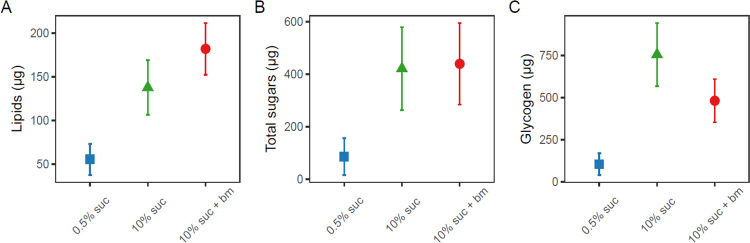
Concentrations (μg) per mosquito of lipids (A), total free sugars (B) and glycogen (C) in individual mosquitoes under differing feeding treatments on day 10 post-emergence. Nutritional perturbations began on day 1 post-emergence and all treatment groups received a blood meal on day 3 post-emergence (with an additional blood meal given to the 10% suc + bm group on day 6). Data presented are estimated marginal means ± SEM.

Mosquitoes fed on the lowest concentration of sucrose (0.5%) had significantly lower lipid levels (55.5±17.8μg) (${\text{X}}_{2}^{2}=17.6$, p < 0.001), approximately 2.5-fold lower than those fed with 10% sucrose (138±31.4μg) and 3.3-fold lower those fed an additional blood meal (182±29.6μg) (Fig. 2A, Table 1). A similar pattern was observed for total sugars: mosquitoes fed with 0.5% sucrose had the lowest total sugar levels (85.6±69.9μg) (${\text{X}}_{2}^{2}=30.8$, p < 0.001), approximately 5-fold lower than those given 10% sucrose (420±158μg) and an additional blood meal (439±156μg) (Fig. 2B, Table 1). Finally, mosquitoes fed with 0.5% sucrose had the lowest glycogen levels (105±64.2μg) (${\text{X}}_{2}^{2}=21.7$, p < 0.001), approximately 7-fold lower than those given 10% sucrose (754±187μg), with mosquitoes given an additional blood meal exhibiting intermediate levels (481±128μg) (Fig. 2C, Table 1).

### Resource availability affects host seeking tendency and biting time of day

**Figure 3.**
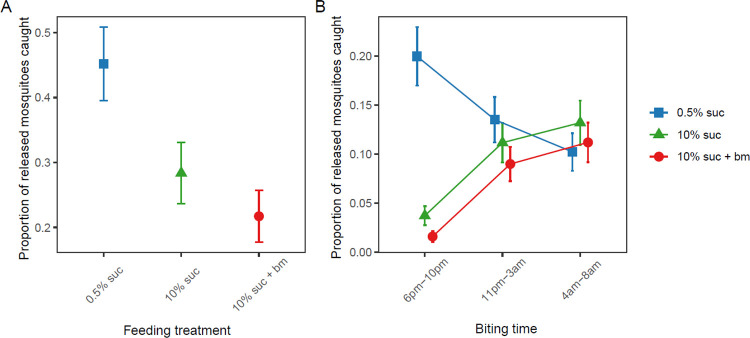
The proportion of released mosquitoes from each feeding treatment that were trapped across the entire night (A) and that were caught in the evening (early, 6pm-10pm), classical night time biting window (11pm-3am) and morning (late, 4am-8am) biting (B). Data presented are estimated marginal means ± SEM.

Across all six releases and trapping times of day, 32.2% of mosquitoes (506/1569) were caught overall. The likelihood of host seeking correlated negatively with the level of nutritional resources provided (${\text{X}}_{2}^{2}=65.8$, p < 0.001) (Fig. 3A). Specifically, mosquitoes fed 0.5% sucrose were 2-fold more likely to be trapped than those fed 10% sucrose (odds ratio (OR): 2.08±0.28) and 3-fold more likely than those fed an additional blood meal (OR: 2.97±0.42). Mosquitoes fed only 10% sucrose were 1.4-fold more likely to be trapped than those fed an additional blood meal (OR: 1.43±0.21).

We also found that nutritional resources differentially affected the time of day of host seeking (${\text{X}}_{4}^{2}=95.4$, p < 0.001). The majority of early biting mosquitoes were from the 0.5% sucrose group, whereas mosquitoes with more resources were more likely to be caught in subsequent traps (Fig. 3B). Specifically, mosquitoes fed 0.5% sucrose were approximately 5- and 10-fold more likely to be caught in the early biting window (20±2.9%) than those fed with 10% sucrose (3.7±0.9%) and an additional blood meal (1.6±0.5%), respectively. Mosquitoes fed 10% sucrose with or without an additional blood meal followed similar temporal patterns, with approximately 4-fold more of these mosquitoes being trapped during the classical (10% sucrose: 11±2.0%, 10% sucrose + bm: 9.0±1.7%) and late biting windows (10% sucrose: 13±2.3%, 10% sucrose + bm: 11±2.0%) than during the early biting window (10% sucrose: 3.7±0.9%, 10% sucrose + bm: 1.6±0.5%).

## Discussion

By altering sugar provision and access to blood meals, and using traps that mimic human breath and scent, we find that resource availability modulates the time of day that mosquitoes search for a blood meal. Mosquitoes on a low sucrose diet had significantly lower levels of lipids, glycogen and sugar, and were 5–10 fold more likely to host seek in the evening (early) than mosquitoes on a high sucrose diet with or without a blood meal (Fig. 2, 3B). In contrast, better-resourced mosquitoes were most likely to host seek during the classical biting time window and into the morning (Fig. 2, 3B). We also find that as resources increased, the overall tendency to host seek decreased; mosquitoes on a low sucrose diet were 2-fold more likely to be caught compared to mosquitoes given a higher concentration of sucrose, and 3-fold more likely than those with access to 10% sucrose and an additional blood meal (Fig. 3A).

Foraging at unusual times of day can be detrimental because it exposes organisms to rhythmic environmental risks, such as the active phase of predators and suboptimal temperature/humidity. Furthermore, circadian rhythms anticipate rhythmic events such as feeding and prepare metabolism accordingly, so foraging at unanticipated times of day may cause suboptimal digestion and nutrition acquisition [1]. However, when resource availability is low, finding food becomes more critical for survival, outweighing the environmental risks and physiological costs [22,25]. For example, a poorly-resourced mosquito may need to become active earlier in the night because they do not have the energy reserves to be able to wait until the classical time of day to forage for blood. In addition, a mosquito that has been unsuccessful at foraging throughout the night may have used significant energy, causing it continue to forage later in the morning because it does not have sufficient reserves to survive until the subsequent night. Our results are consistent with our hypothesis for early biting because poorly-resourced mosquitoes with low nutritional reserves are more likely to host seek, predominantly in the early evening when temperatures are higher and humidity lower, and risk being killed because human hosts are alert. Although we did not quantify survival, we did not encounter any live mosquitoes fed on a 0.5% sucrose diet whilst clearing the semi-field systems the morning after each release, but regularly encountered live mosquitoes fed on higher sucrose solutions and/or an additional blood meal. Overall, this suggests that early biting is an adaptive temporal shift, which may be necessary in regions which are sugar-poor and hosts are not easily accessible due to high ITN use, and/or where larvae are poorly nourished.

That better-resourced mosquitoes on high sucrose diets (with or without a blood meal) had higher nutritional reserves and waited until the classical time window to host seek, when environmental risks are likely to be lower, also supports our hypotheses. However, we also found that better-resourced mosquitoes were just as likely as poorly resourced mosquitoes to be trapped later into the morning. Such late biting by well-fed mosquitoes was unexpected but may be due to the lack of water/food sources provided in the semi-field enclosures, forcing untrapped mosquitoes to host seek to mitigate dehydration caused by the rise in temperature and the reduction in humidity in the morning [48,49]. Alternatively, this finding may be explained by our hypothesis that well-resourced mosquitoes which had failed to blood feed (*i.e*. had not entered a trap) during the night have become resource-depleted, leading to an increased propensity to continue host seeking at unusual times of day. Regardless of the explanation(s), our results reveal that adult nutritional condition drives variation in biting time of day.

To probe the ecological relevance of our experimental results, we also undertook a trapping session for wild mosquitoes (see Supplementary Information). While the nutritional content of the 66 *Anopheles* spp. mosquitoes we caught between 6pm and 7am varied, levels were not significantly different to the profiles of low-resourced (0.5% sucrose) experimental mosquitoes (Supplementary Fig. S3, Table S1). Our low sample size precludes comparing nutritional reserves of wild mosquitoes caught host seeking at different times of day, but suggests that adult nutritional content does not correlate with body size (a product of larval condition) (Fig. S4). Thus, catching lowly resourced mosquitoes at the time of host seeking is consistent with our experimental results revealing that adult nutrition influences biting rhythms. Furthermore, like previous studies across Africa [3,50,51], we found a large proportion (approximately 20%) of ‘late’ biting occurring in the morning when humans are unprotected by bed nets. This pattern has become more pronounced since the widespread use of ITNs. Because biting time shifts have a genetic component [20], and universal coverage of ITNs is approximately 75% in Ghana [52], late biting could be an evasion strategy evolving in the mosquito population where the larval collections for our experiment were carried out, rather than a plastic behavioural change. However, further work is needed to ascertain the extent to which biting time of day is genetically determined and/or due to behavioural plasticity in response to variation in resource availability. For example, modelling suggests that early biting is more likely to evolve in sugar-poor environments than sugar-rich environments [28]. However, in areas of fluctuating resource availability or ITN coverage, plastic genotypes that can switch between early, classical, or late biting depending on their environmental conditions should have higher fitness [21].

Both our semi-field experiment and preliminary field data demonstrate significant variation in biting time of day. Parasite fitness is a culmination of the parameters contributing to R_0_, many of which are directly related to within-vector parasite development, vector survival and vector behaviour [16]. Intuition suggests that early biting by poorly-resourced mosquitoes could sustain malaria transmission. However, the overall impact of shifts in biting time of day for malaria transmission is difficult to predict, because of potential synergistic and antagonistic interactions between R_0_’s constituent parameters. For example, while early biting causes greater contact between vectors and hosts in regions with high ITN coverage, low-resourced mosquitoes have reduced survival [45]. Thus, while early biters are more likely to obtain a blood meal, their poor nutritional state could curtail onward transmission if vectors do not survive long enough for malaria parasites to complete their development [53,54]. Furthermore, if early biters are more likely to be low-resourced mosquitoes, there are conflicting outcomes for parasites. While early biting is more suitable for parasite establishment in the mosquito due to lower night-time temperatures [55] and higher infectivity than in the day time [1,17,19], parasite development is slower and less productive in low-resourced mosquitoes [45,56,57]. In addition, disruption to foraging rhythms reduces fitness in other insects [58], suggesting that blood feeding at a non-classical time of day may be suboptimal for mosquitoes. The breakdown of blood meal contents leads to high levels of reactive oxygen species (ROS), and mosquitoes have an anticipatory rhythmic detoxification mechanisms for coping with oxidative stress [59]. Thus, if blood feeding at an unusual time of day causes detoxification mechanisms and metabolic processes to be mismatched to the time of day blood is digested, mosquitoes may experience higher oxidative stress and/or less productive digestion of nutrients, both of which would reduce parasite fitness. While transmission at a non-classical time of day is clearly better for parasites than no transmission at all in areas of ITN use, recognising that transmission is time of day dependent is necessary for accurately predicting epidemiology, the risk of clinical malaria cases, and the trajectory of parasite evolution.

## Conclusions and Implications

Our results suggest that plasticity in biting time of day, driven by variation in adult mosquito nutritional condition, is an underappreciated contributor to residual malaria transmission. Addressing the drivers of variation in biting time of day and the overall impact on malaria transmission is critical for assessing and improving the efficacy of current and future vector control tools harnessing mosquito foraging behaviours. While this is especially important for malaria, where up to 30% of biting is reported to occur when humans are unprotected by bed nets and is predicted to lead to millions of additional clinical cases per year [3,10], we also expect our findings to apply to other vector-borne diseases that rely on vector foraging rhythms for between-host transmission.

## Supplementary Material

Supplement 1

## Figures and Tables

**Table 1.** Post hoc pairwise comparisons for nutritional content of mosquitoes provided with different feeding treatments. Significant p-values (<0.05) are highlighted in bold, and borderline p-values (0.05<p<0.09) are italicised and underlined.

		Test statistic	p-value
**Lipids**	0.5% suc – 10% suc	*t = 2.75*	**0.02**
	0.5% suc – 10% suc + bm	*t = −4.54*	**<0.001**
	10% suc – 10% suc + bm	*t = −1.16*	0.48
**Total sugars**	0.5% suc – 10% suc	*t = 5.13*	**<0.001**
	0.5% suc – 10% suc + bm	*t = −6.13*	**<0.001**
	10% suc – 10% suc + bm	*t = −0.21*	0.98
**Glycogen**	0.5% suc – 10% suc	*t = 4.66*	**<0.001**
	0.5% suc – 10% suc + bm	*t = −3.69*	**0.002**
	10% suc – 10% suc + bm	*t = 1.55*	0.28
