# Supplementary material for: Biting time of day in malaria mosquitoes is modulated by nutritional status
Source: bioRxiv. 2025 Aug 18:2025.04.28.650966. Originally published 2025 May 1. Preprint. [Version 2] doi: 10.1101/2025.04.28.650966 (PMC12247726; doi:10.1101/2025.04.28.650966)
Supplement: Supplement 1 [file media-1.pdf]

Supplementary Figures

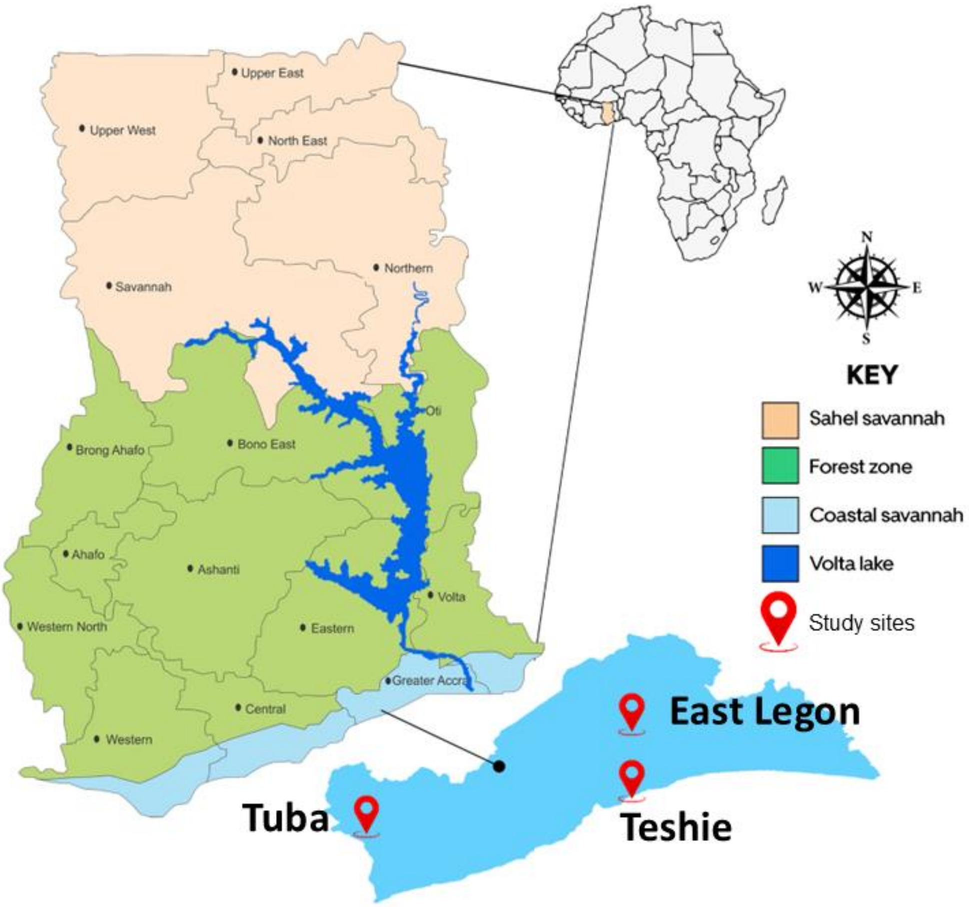

Figure S1. Map of larval and adult mosquito collection sites in Greater Accra, Ghana. Larvae from the three sites were collected in Dec 2023-Jan 2024, combined, and female F_2 progeny were used for mosquito nutrition perturbations and biting time of day assays. Wild adult mosquitoes were collected from Teshie across one night.

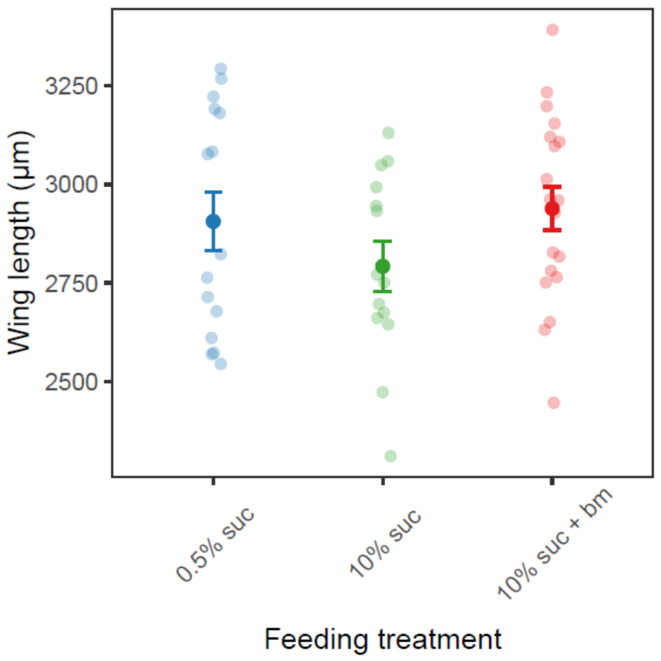

Figure S2. Wing length (a proxy for body size) of lab-reared mosquitoes under different feeding treatments. Data presented are means ± SEMs, with each point representing an individual mosquito. While mosquito size differed between release batches ($F_{2,45}$=5.55, p=0.007), this was consistent across feeding treatments within each batch ($F_{3,40}$=0.05, p=0.98). Overall, there was no difference between wing length across feeding treatment groups ($F_{2,43}$=0.66, p=0.52).

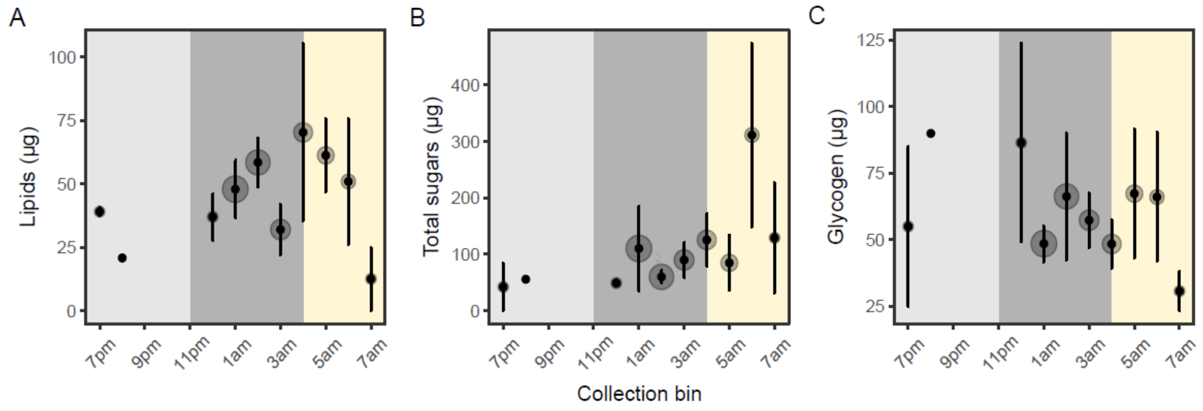

Figure S3. Concentrations (µg) per mosquito of lipids (A), total free sugars (B) and glycogen (C). Data presented are means ± SEM, and the size of the grey circle reflects the sample size per hourly bin.

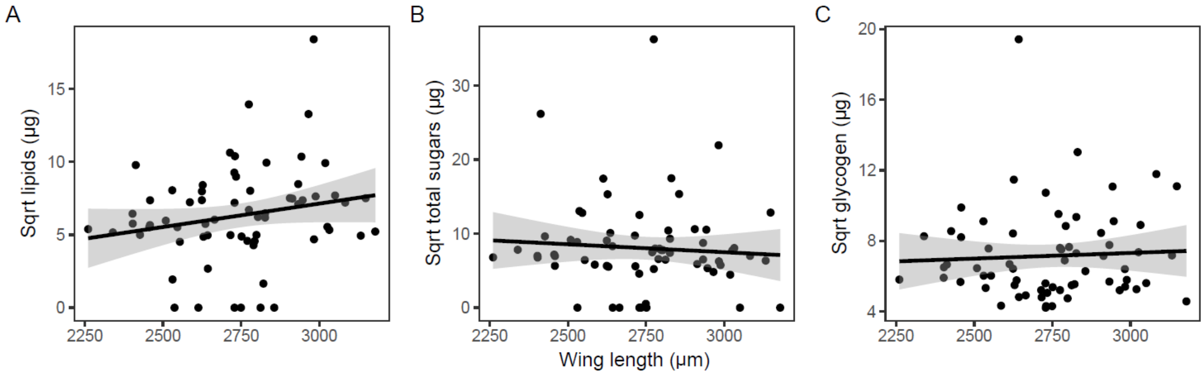

Figure S4. The relationship between wing length (a proxy for body size) and (A) lipids, (B) total free sugars and (C) glycogen in wild-caught adult mosquitoes. Data points represent an individual mosquito, with lines and shading denoting the best fit linear model estimates and 95% confidence intervals. For lipids ($F_{(1,64)}$ = 2.80, p = 0.10), total free sugars ($F_{(1,64)}$ = 0.35, p = 0.56) and glycogen ($F_{(1,64)}$ = 0.18, p = 0.67), there was no correlation with wing length.
