# Supplementary material for: Biting time of day in malaria mosquitoes is modulated by nutritional status
Source: bioRxiv. 2025 May 1:2025.04.28.650966. Preprint. [Version 1] doi: 10.1101/2025.04.28.650966 (PMC12247726; doi:10.1101/2025.04.28.650966)
Supplement: Supplementary file 1 [file media-1.pdf]

Supplementary Information

Supplementary Methods

Wild mosquito collections

To investigate biting time of day and nutritional content in wild mosquitoes, we collected adult mosquitoes (N=95) from one site in the Greater Accra region of Ghana (Teshie, 5° 35′ 0″ N, 0° 6′ 0″ W) (Supplementary Fig. S1) overnight between 6pm and 7am in February 2024, using the human landing catch (HLC) technique. This technique involves trained human collectors exposing their lower legs and collecting mosquitoes that land on them before they bite, and is the gold standard for investigating human exposure to disease vectors [1]. Every hour, we snap-froze collected mosquitoes on dry ice and identified specimens to genus level according to morphological characteristics [2], noting the number of *Anopheles* spp. mosquitoes collected per hourly bin. We placed individual anopheline mosquitoes in 1.5ml microcentrifuge tubes and stored them on dry ice, transferring them to a -20°C freezer after the overnight collection period had ended.

Nutrition assays

We quantified mosquito nutritional status in N=66 wild-caught mosquitoes individually to assess whether our experimental diets generated mosquitoes with ecologically relevant levels of resources, by conducting nutrition assays as described in the main text, except 250µl supernatant was used for total sugars analysis.

Additional statistical analysis

We conducted preliminary analysis to see how nutritional content between field-caught and lab-reared mosquitoes varied, using linear models to compare between the field-caught, 0.5% suc, 10% suc and 10% suc + bm groups. We performed a chi-square test to investigate if the number of field-caught mosquitoes differed throughout the night by grouping hourly bins into 'early' biters (6pm-11pm), 'classical' biters (11pm-4am) and 'late' biters (4am-7am). Using linear models, we analysed the nutritional content (lipid, glycogen, total sugars) of individual wild-caught mosquitoes with biting time of day as an unordered factor, and assessed the correlation between wing length and nutritional content. Nutrition data were square root transformed to meet assumptions of normality and homogeneity of variance. We conducted post hoc pairwise comparisons using the Tukey method with the *emmeans* package to compare nutritional content between field-caught and lab-reared mosquitoes.

References

1. Gimnig JE, Walker ED, Otieno P *et al.* Incidence of Malaria among Mosquito Collectors Conducting Human Landing Catches in Western Kenya. *The American Journal of Tropical Medicine and Hygiene* 2013;**88**:301–8.

2. Gillies MT, Coetzee M. A Supplement to the Anophelinae of Africa South of the Sahara. *Publications of the South African Institute for Medical Research* 1987;**55**:1–143.

Supplementary Figures

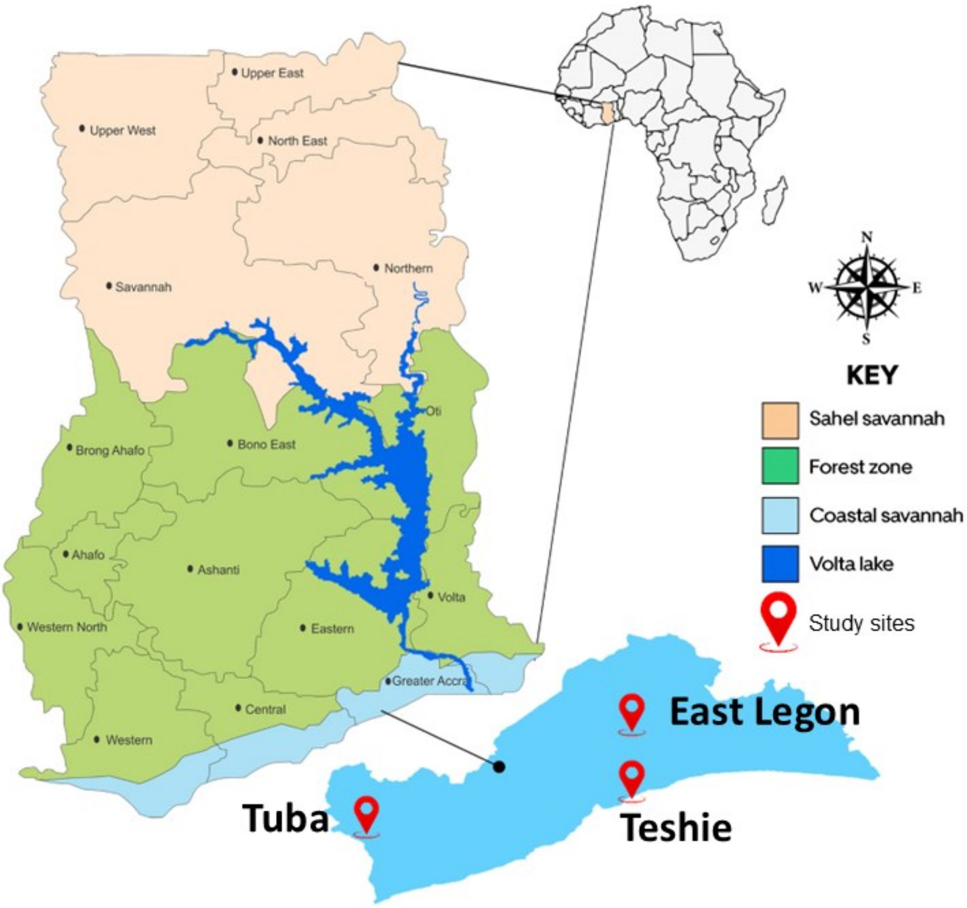

Figure S1. Map of larval and adult mosquito collection sites in Greater Accra, Ghana. Larvae from the three sites were collected in Dec 2023-Jan 2024, combined, and female F_2 progeny were used for mosquito nutrition perturbations and biting time of day assays. Wild adult mosquitoes were collected from Teshie across one night.

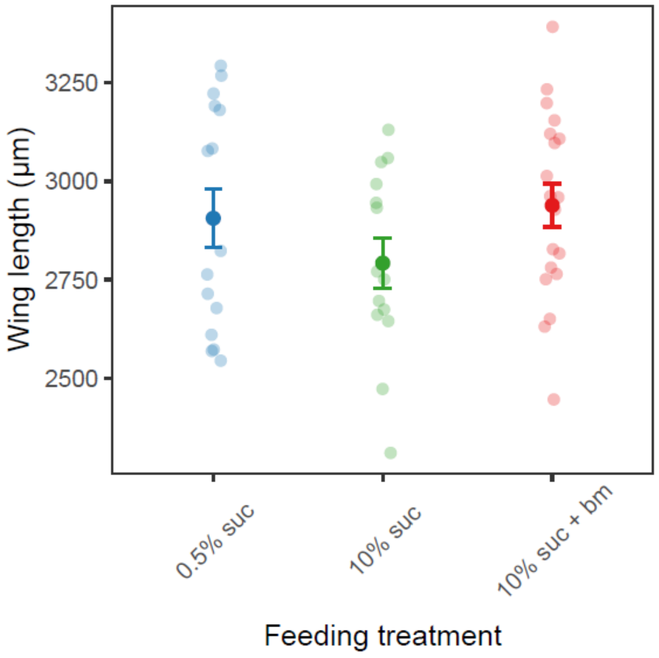

Figure S2. Wing length (a proxy for body size) of lab-reared mosquitoes under different feeding treatments. Data presented are means ± SEMs, with each point representing an individual mosquito. While mosquito size differed between release batches ($F_{2,45}$=5.55, p=0.007), this was consistent across feeding treatments within each batch ($F_{3,40}$=0.05, p=0.98). Overall, there was no difference between wing length across feeding treatment groups ($F_{2,43}$=0.66, p=0.52).

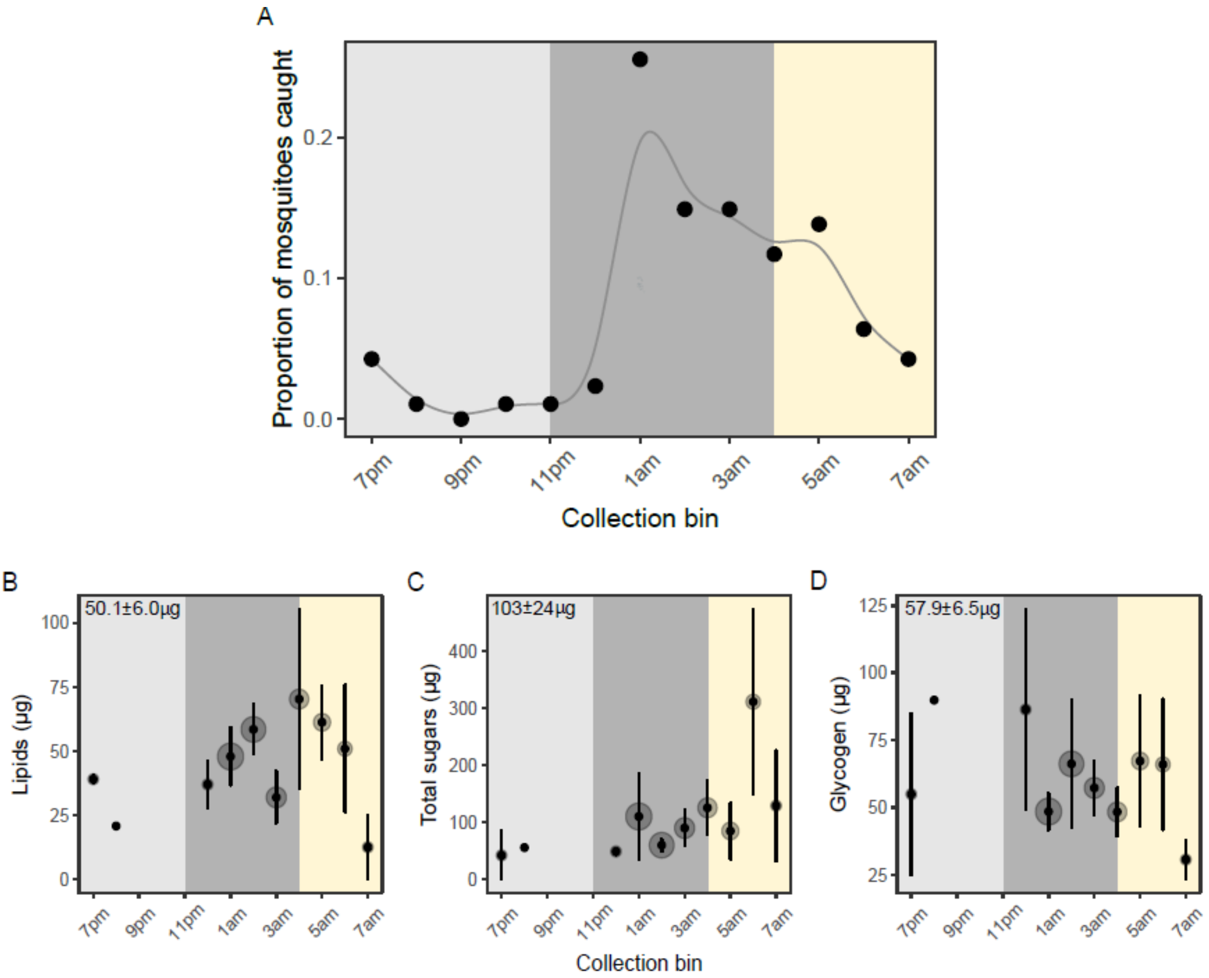

Figure S3. The proportion of wild adult *Anopheles* spp. mosquitoes caught by HLC collections across hourly bins between 6pm and 7am (A), and their body size adjusted concentrations (µg) per mosquito of lipids (B), total free sugars (C) and glycogen (D). Early, classical, and late bins are indicated by shaded regions from left to right. To highlight how the pattern changes over the course of the night, data points in (A) are connected by an X-spline. In (A) we caught a total of 95 mosquitoes, the majority of which were caught biting during the classical window and peaked between 12am-1am (A). We grouped hourly bins into 'early' biters (6pm-11pm), 'classical' biters (11pm-4am) and 'late' biters (4am-7am) and found that there were differences in the number of mosquitoes caught across these windows ($\chi^2_{(2)} = 45.0$, $p < 0.001$). Specifically, 68% of mosquitoes were caught during the classical biting window, compared to 7% and 24% caught during the evening and morning windows respectively. Data presented in (B)-(D) are means ± SEM, and the size of the grey circle reflects the sample size per hourly bin. While mosquitoes varied in nutritional content, there were no differences between lipid ($F_{(9,56)} = 0.75$, $p = 0.66$), sugars ($F_{(9,56)} = 1.06$, $p = 0.40$) or glycogen ($F_{(9,56)} = 0.40$, $p = 0.93$) contents across hourly bins. The overall mean ± SEM for each nutrition type is displayed in the top left of each graph.

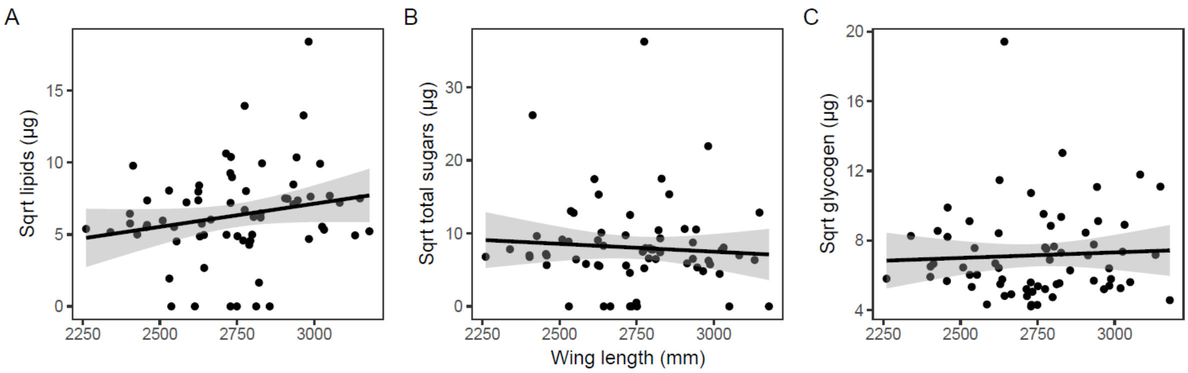

Figure S4. The relationship between wing length (a proxy for body size) and (A) lipids, (B) total free sugars and (C) glycogen in wild-caught adult mosquitoes. Data points represent an individual mosquito, with lines and shading denoting the best fit linear model estimates and 95% confidence intervals. For lipids ($F_{(1,64)} = 2.80$, $p = 0.10$), total free sugars ($F_{(1,64)} = 0.35$, $p = 0.56$) and glycogen ($F_{(1,64)} = 0.18$, $p = 0.67$), there was no correlation with wing length.

Supplementary Tables

Table S1. Pairwise comparisons of the nutritional content of field-caught mosquitoes versus. lab-reared mosquitoes on different feeding treatments. Significant p-values (<0.05) are highlighted in bold.

		Test statistic	***p-value***
	field – 0.5% suc	*t* = -1.25	0.59
**Lipids**	field – 10% suc	*t* = -4.82	**<0.001**
	field – 10% suc + bm	*t* = -7.91	**<0.001**
	field – 0.5% suc	*t* = -1.13	0.67
**Total sugars**	field – 10% suc	*t* = -4.95	**<0.001**
	field – 10% suc + bm	*t* = -8.06	**<0.001**
	field – 0.5% suc	*t* = -1.96	0.21
**Glycogen**	field – 10% suc	*t* = -11.3	**<0.001**
	field – 10% suc + bm	*t* = -8.86	**<0.001**
